# Supplementary material for: Compositional Data Analysis using Kernels in mass cytometry data
Source: Bioinform Adv. 2022 Feb 11;2(1):vbac003. doi: 10.1093/bioadv/vbac003 (PMC8867823; doi:10.1093/bioadv/vbac003)
Supplement: vbac003_Supplementary_Materials [file vbac003_supplementary_materials.pdf]

Supplementary Materials for Compositional Data Analysis using Kernels in Mass Cytometry Data

Pratyaydipta Rudra, Ryan Baxter, Elena Hsieh, Debashis Ghosh

S1 Choice of Kernels and Permutation Methods

Currently, CODAK provides 3 choices of distances for the kernel measuring similarity of the compositional profiles. These options are:

1. Aitchison Distance (AD) (default)
2. Bray-Curtis Distance (BCD)
3. Euclidean Distance (ED)

CODAK using these distances are denoted by CODAK, CODAK-BC and CODAK-ED in our plots. In each case, we allow the user to choose the kernel using Gower's method (default) as in Equation 3 or the Gaussian kernel as in Equation 6. Based on our empirical results, we suggest using CODAK with the default choices (See Figures 3, S2 and S6).

For the predictor variable, CODAK allows the user to choose either the linear kernel (LK) or the Gaussian kernel (GK). Based on our empirical results, we suggest using the LK (See Figure S7).

Three choices of permutation methods are available when adjusting for covariates using the CODAK-alr method. These are:

1. Shuffle residuals (KC) (Kennedy and Cade, 1996): Residualize both Y and X with respect to the covariates and permute the residuals.
2. Shuffle Y (SY) (Manly, 2018): Shuffle the rows of the Y matrix while keeping the relationship between X and Z intact.
3. Freedman and Lane's method (FL) (Freedman and Lane, 1983): Permute residualized data, then add the estimated covariate effects back, and fit model again.

See Anderson and Legendre (1999) for detailed description of each permutation method and the subtle differences between them. It is well known that the SY method is less stable than the other two methods and it can perform poorly in the presence of outliers (Anderson and Legendre, 1999; Winkler *et al.*, 2014). Therefore, we recommend using one of the other two methods. Our empirical results show that the FL method has the best performance since it controls the type-I error well. The KC method has higher power, but it is also slightly anticonservative (Figure 5, Figure S4).

S2 Additional Discussion on the relationship between the R^2 measures

As discussed in Section 2.6, the R^2 provided by MiRKAT (Zhao *et al.*, 2015) is the square of the dcor measure (Equation 15) in CODAK when using the same kernel for the compositional data and LK for the predictor. The R^2 provided by PERMANOVA (Anderson, 2001) is not equivalent to these. Suppose we use the LK for the predictor and AD kernel for the compositions. The kernel matrix K for the compositions is obtained using Equation 3. Note that the R^2 measure for PERMANOVA is given by (Anderson, 2014)

$$R^2 = \frac{trace(H_X K)}{trace(K)}, \tag{S1}$$

where H_X is the hat matrix defined using X. On the other hand, the dcor for CODAK is given by

$$dcor(P, X) = \frac{\text{trace}(KHXX'H)}{\sqrt{\text{trace}(KHKH)\ \text{trace}(XX'HXX'H)}}. \tag{S2}$$

Although it can be shown that the two expressions become equivalent when P is one-dimensional, it is not useful in our case since the compositional profile P is not one-dimensional here. We provide an empirical comparison of the R^2 measures from the 3 methods (CODAK, MiRKAT-AD, PERMANOVA-AD) using simulations in the bottom panels of Figure S1. The top panels of this figure shows the equivalence of three methods in the no covariate case, confirming the theoretical results in Hua and Ghosh (2015) and Pan (2011). The near perfect association in the top panels shows that the methods are equivalent in these cases and the only difference is likely due computation. The simulations were conducted using the simulation scheme to produce Figure 3.

S3 Computation Time

We compared the computation time for each of the commonly used methods to test cell type abundance in mass cytometry on a single machine with i7-10700 CPU @2.90 GHz and 32GB RAM. For each method, we ran it 100 times on a typical data set as obtained from our simulation schemes using 1000 permutations. The run times are reported in Table S1.

Table S1: Table showing computation time for 100 simulations. Number of permutations used = 1,000.

Method	Time (seconds)
CODAK	2.91
GLMM	131.15
diffcyt	3.75
GLMM (permutation)	130450.8

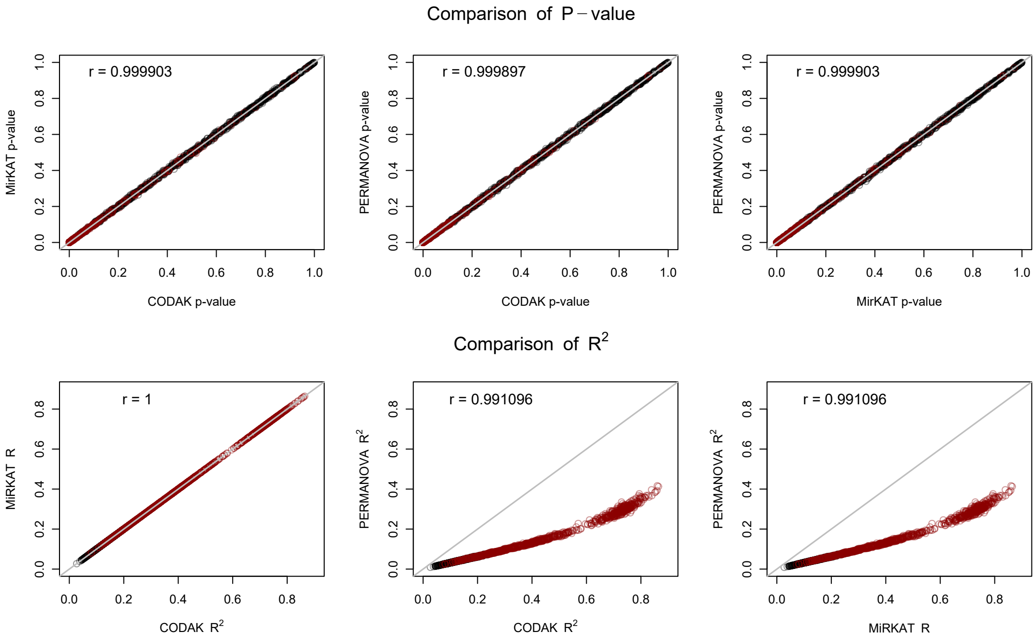

Figure S1: Scatter plot comparing the p-values (top panels) and R^2-like measures (bottom panels) from CODAK, PERMANOVA and MiRKAT in the no covariate case from simulated data sets. AD kernel and linear kernel were used for the compositions and the predictor variable, respectively. Black dots are from the null scenario (no association), and red dots are from the alternative (some association). The near perfect association in the top panels and the bottom left panel shows that the methods are equivalent in these cases and the only difference is likely due computation.

S4 Additional Simulations

S4.1 Simulation details

We conducted the following simulation studies besides the ones reported in the main manuscript.

S(i) Continuous predictor (Figure S2): This is identical to scenario (i) in the main manuscript (Figure 3) except that the predictor variable is a continuous variable simulated from a $U(0,1)$ distribution. For comparability, the effect sizes in cases (a)-(d) are simulated such that the mean and variance of βX remains same as the binary predictor case, where β is the q-dimensional vector of effect sizes.

S(ii) Binary predictor with larger random effects variance (Figure S3): We also wanted to explore the effect of a larger random effect variance. This simulation is identical to the to scenario (i) in the main manuscript (Figure 3) except that the standard deviation of the random effects term is now assumed to be 0.5.

S(iii) Continuous predictor, binary covariate (Figure S4): This is similar to scenario (ii) in the main manuscript (Figure 5) except that the predictor is now considered to be continuous like S(i) above.

S(iv) Binary predictor, binary covariate, repeated measures with larger dependence (Figure S5): This is similar to scenario (iii) in the main manuscript (Figure 6) except that the dependence across the repeated measures is now larger (random effect SD = 0.3 instead of 0.1).

S(v) Comparison of CODAK (with AD) using default kernel vs Gaussian kernel, binary predictor (Figure S6): The simulation scheme is similar to scenario (i) in the main manuscript (Figure 3).

S(vi) Comparison of CODAK for linear kernel vs Gaussian kernel for the predictor, continuous predictor (Figure S7): The simulation scheme is similar to scenario S(i) above.

S(vii) Comparison of CODAK-ED vs original distance correlation (dcor), continuous predictor (Figure S8): The simulation scheme is similar to scenario S(i) above.

S(viii) Comparison of statistical power (binary predictor) for data with no zeros and data with some zeros each of which is replaced by a pseudocount of 1 (Figure S9).

S4.2 Results of the additional simulations

The comparisons of the size and power for the methods under consideration for simulations S(i)-S(viii) are shown in Figures S2-S9, respectively. The diffcyt methods are not available for the continuous predictor cases.

The findings from Figure S2 and Figure S4 are similar to the simulations in the main manuscript.

The advantage of CODAK over the other two methods is slightly less when a larger random effects variance is used (Figure S3).

The CODAK-sk method is still the most robust among all methods against the violation of the independence assumption in Figure S5 when the violation is stronger, but it gradually starts to become anticonservative as the dependence in the repeated measures increase.

Figure S6 shows that the default kernel and Gaussian kernel using AD have similar performance, with the default kernel performing slightly better. Figure S7 shows that the linear kernel performs better than Gaussian kernel for measuring the predictor similarities.

Figure S8 shows that CODAK-ED and original dcor, although not equivalent, have near identical performance. However, both have poor statistical power, and are not recommended for compositional data.

Figure S9 shows that there is a small amount of power loss for replacing the zeros by pseudocounts when compared to the situation with no zeros.

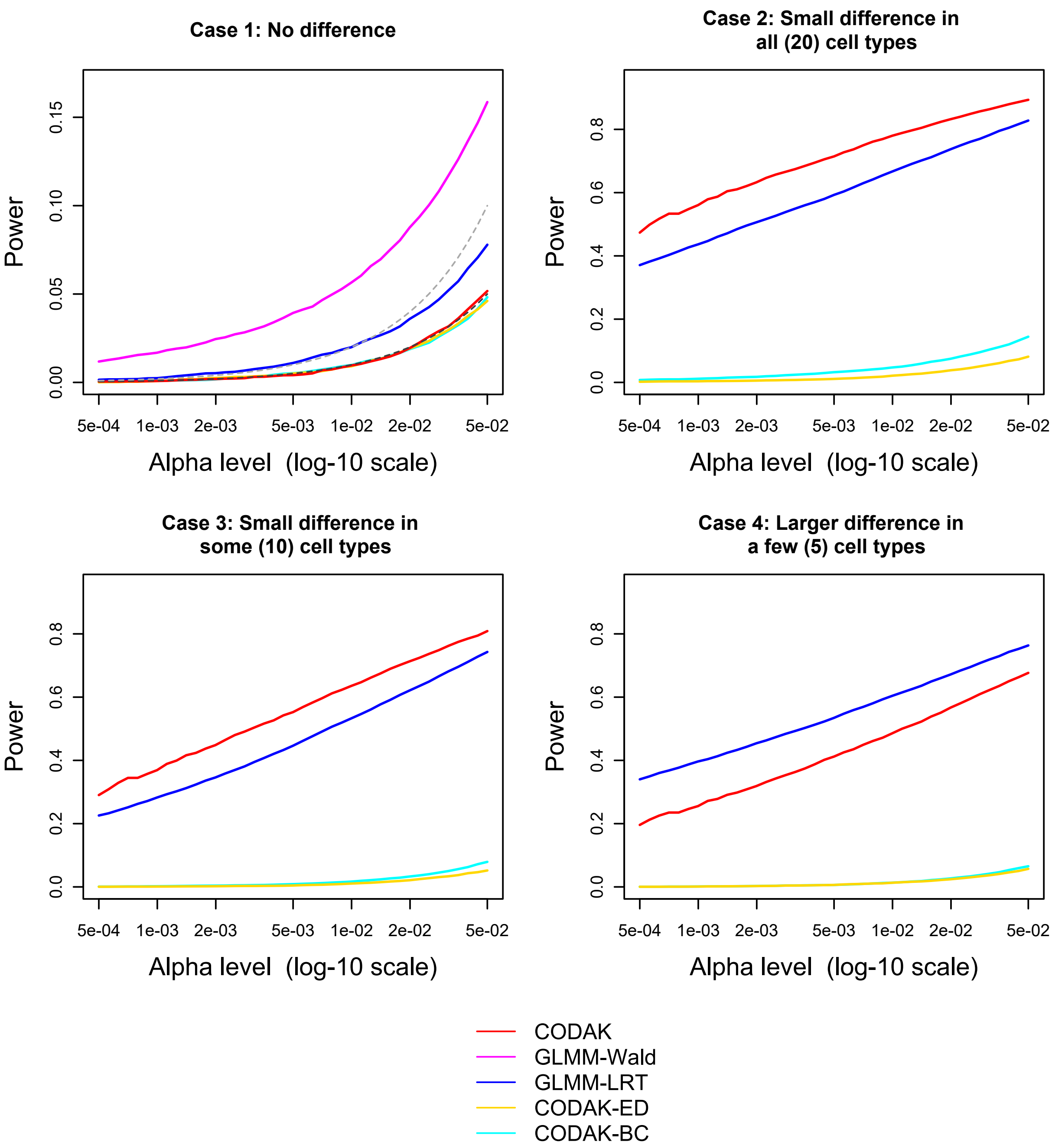

Figure S2: Comparison of statistical power for continuous predictor. The black dashed line in the first plot shows the nominal level α and the grey dashed line shows two times α. Only the methods with reasonable control of type-I error are shown in the other 3 plots.

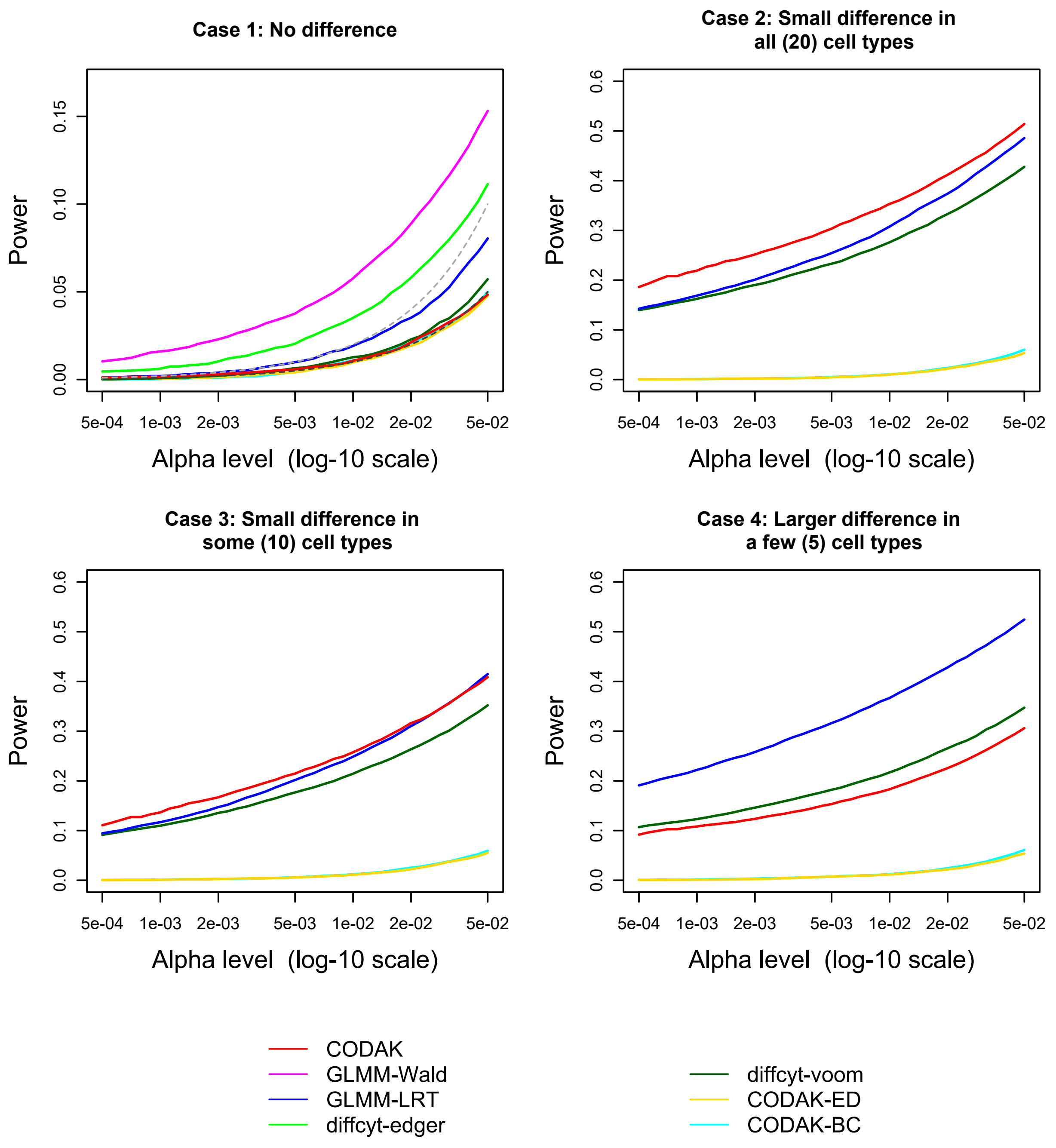

Figure S3: Comparison of statistical power for larger random effects variance (binary predictor). The black dashed line in the first plot shows the nominal level α and the grey dashed line shows 2α. Only the methods with reasonable control of type-I error are shown in the other 3 plots.

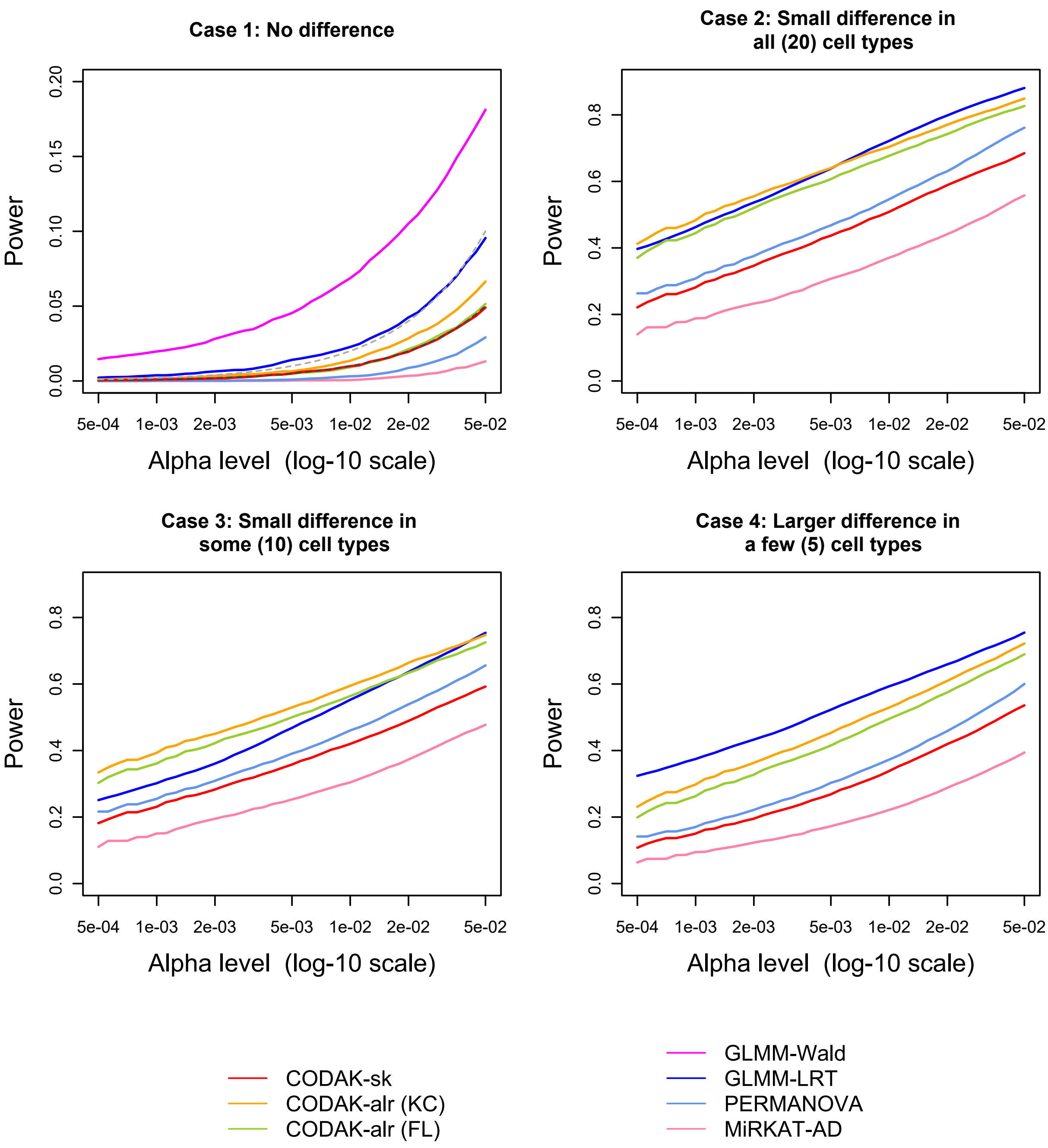

Figure S4: Comparison of statistical power for continuous predictor, categorical covariate. The black dashed line in the first plot shows the nominal level α and the grey dashed line shows 2α. Only the methods with reasonable control of type-I error are shown in the other 3 plots.

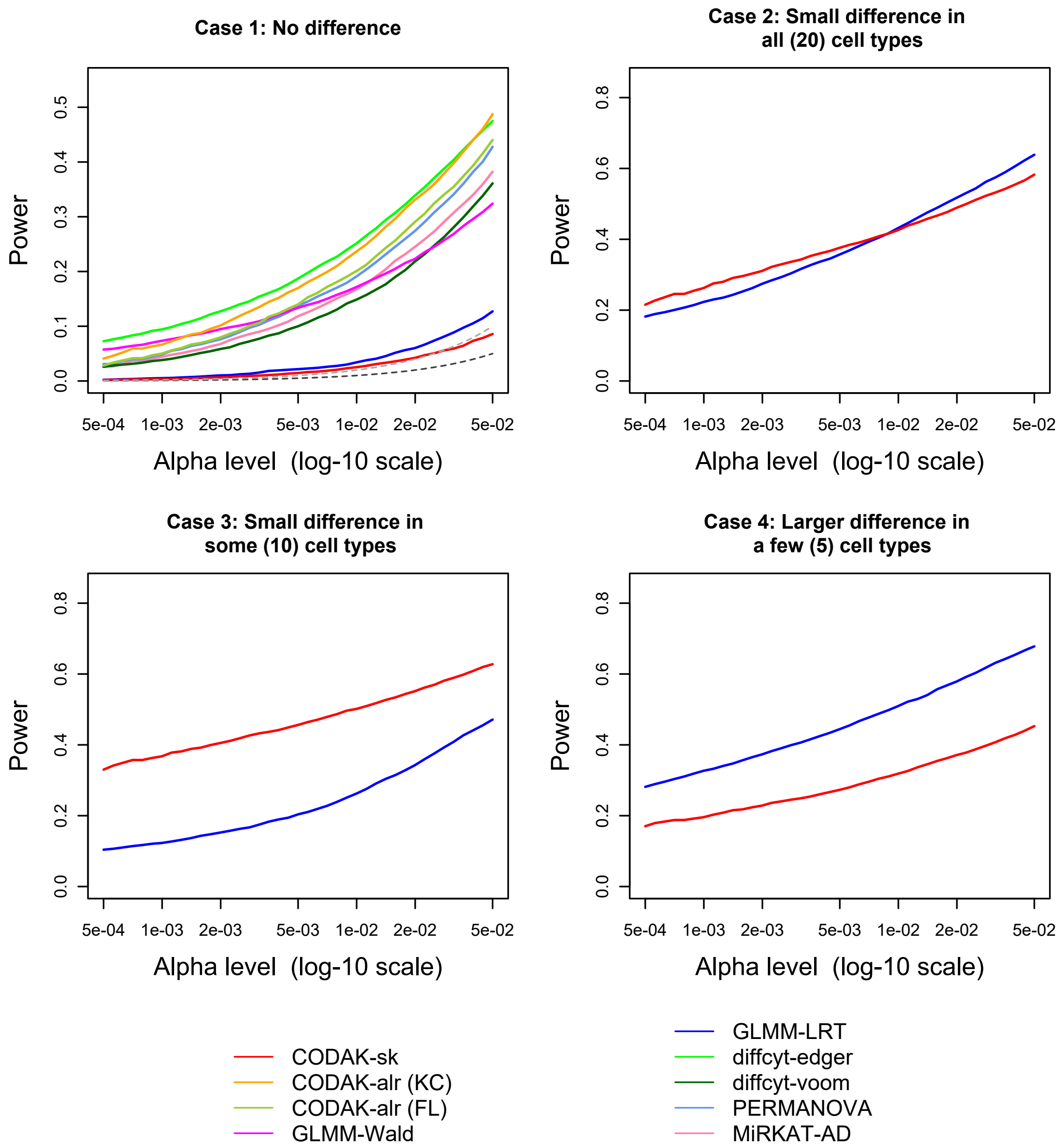

Figure S5: Comparison of statistical power for binary predictor adjusting for a binary covariate with repeated measures when the amount of dependence across repeated measures is high. The black dashed line in the first plot shows the nominal level α and the grey dashed line shows two times α. Only the methods with reasonable control of type-I error are shown in the other 3 plots.

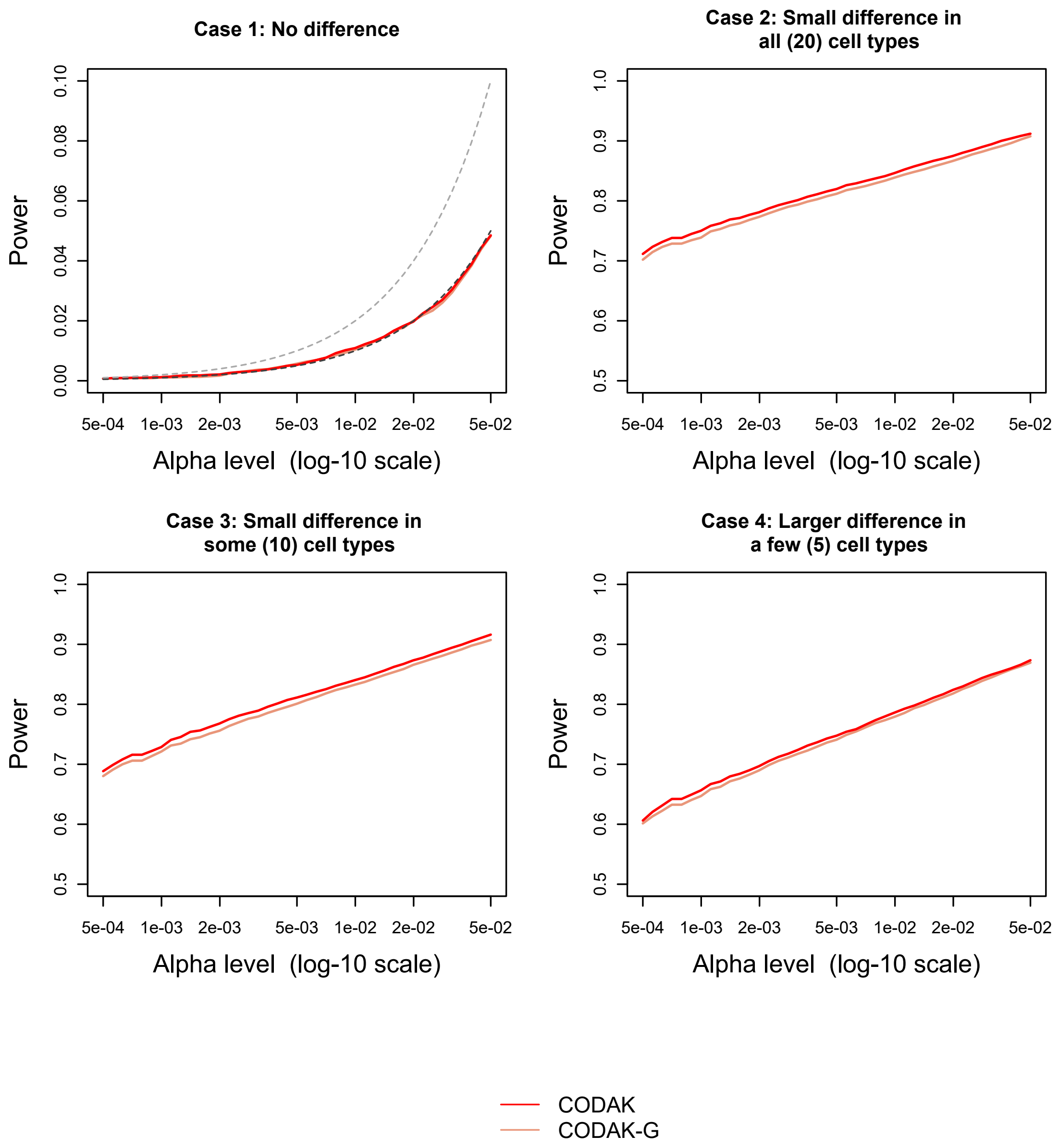

Figure S6: Comparison of statistical power for binary predictor for different choices of the kernel for the compositions; CODAK: AD kernel using Equation 3, CODAK-G: AD kernel using Equation 6. The black dashed line in the first plot shows the nominal level α and the grey dashed line shows 2α.

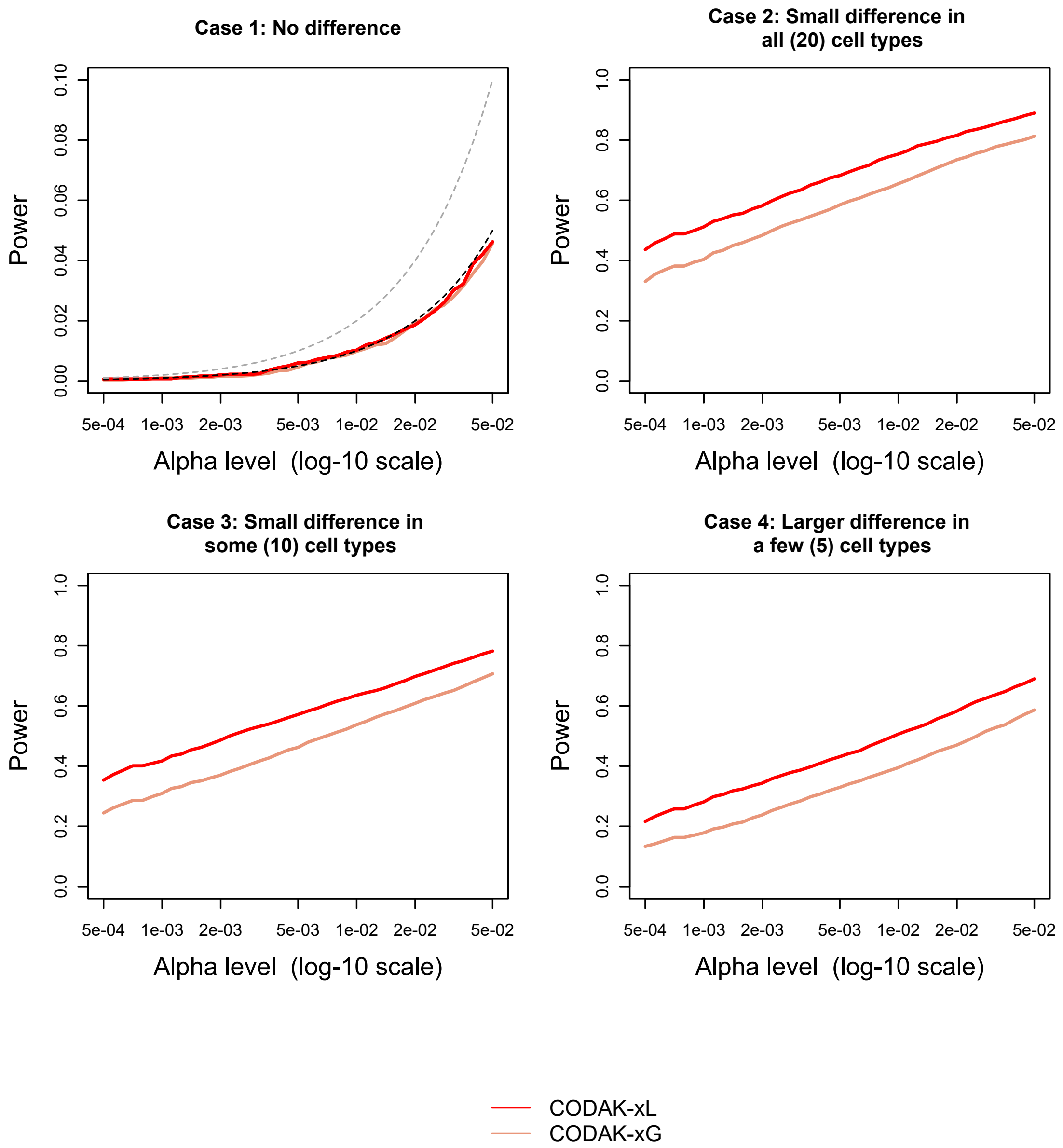

Figure S7: Comparison of statistical power for continuous predictor for different choices of the kernel for the predictor variable; CODAK-xL: linear kernel for the predictor, CODAK-xG: Gaussian kernel for the predictor. The black dashed line in the first plot shows the nominal level α and the grey dashed line shows 2α.

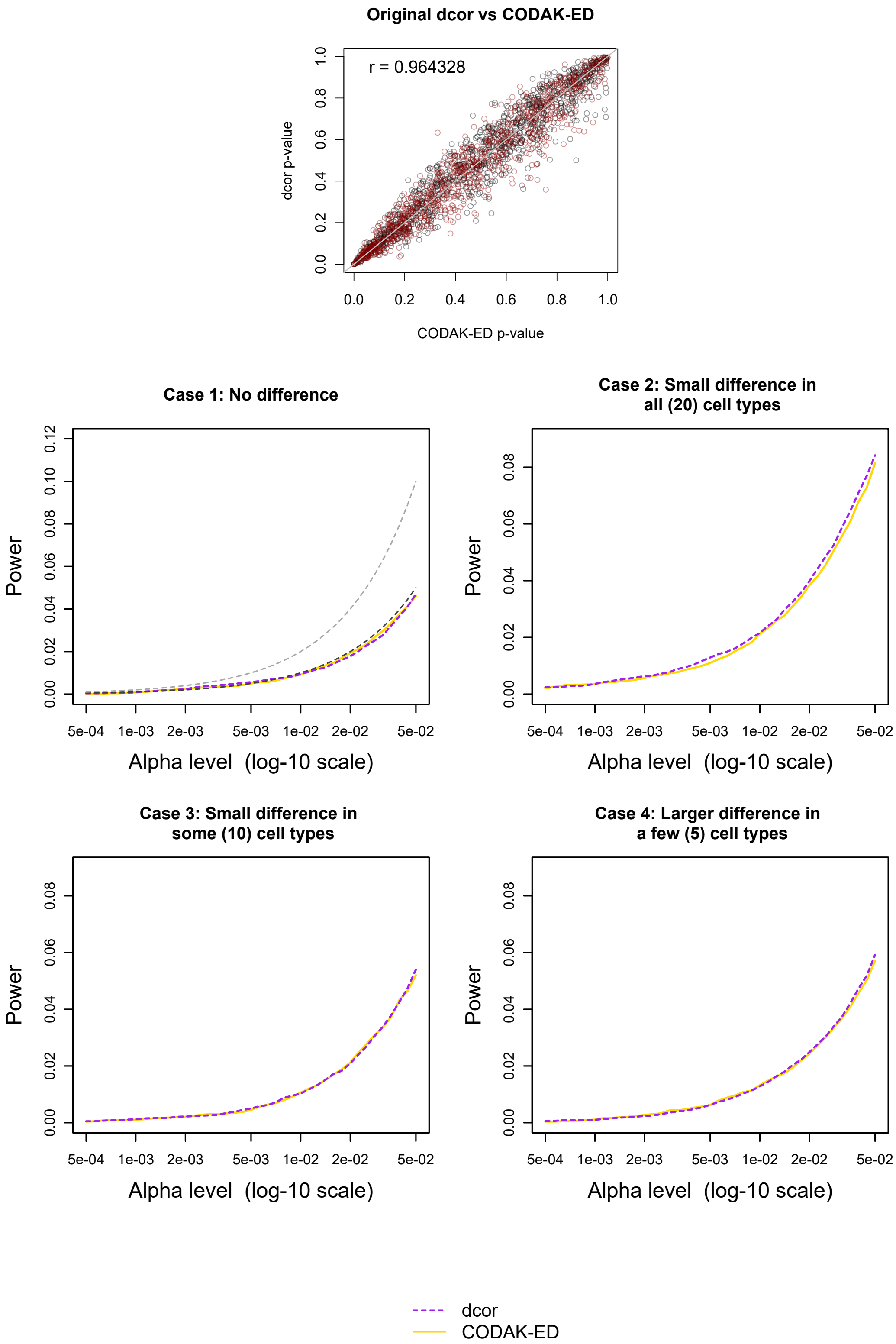

Figure S8: Top panel: Comparison of p-values for original distance correlation and CODAK-ED. Bottom 4 panels: Comparison of statistical power for continuous predictor for CODAK-ED vs the original distance correlation (dcor). The black dashed line in the first plot shows the nominal level α and the grey dashed line shows 2α. Although the power and type-I error are nearly identical, it is clear from the top panel that the two methods are not exactly equivalent.

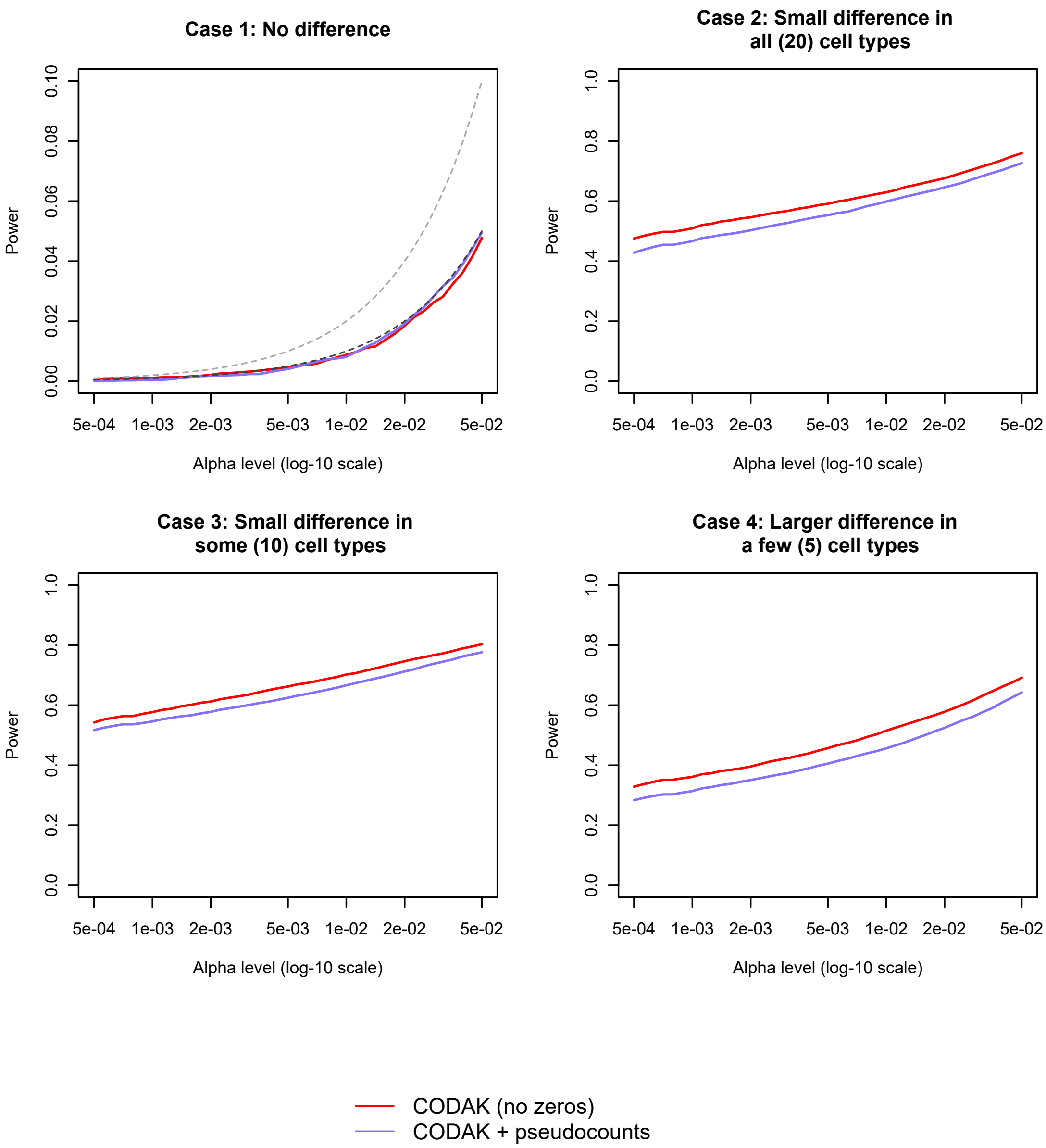

Figure S9: Comparison of statistical power (binary predictor) for data with no zeros and data with some zeros each of which is replaced by a pseudocount of 1. The black dashed line in the first plot shows the nominal level α and the grey dashed line shows 2α.

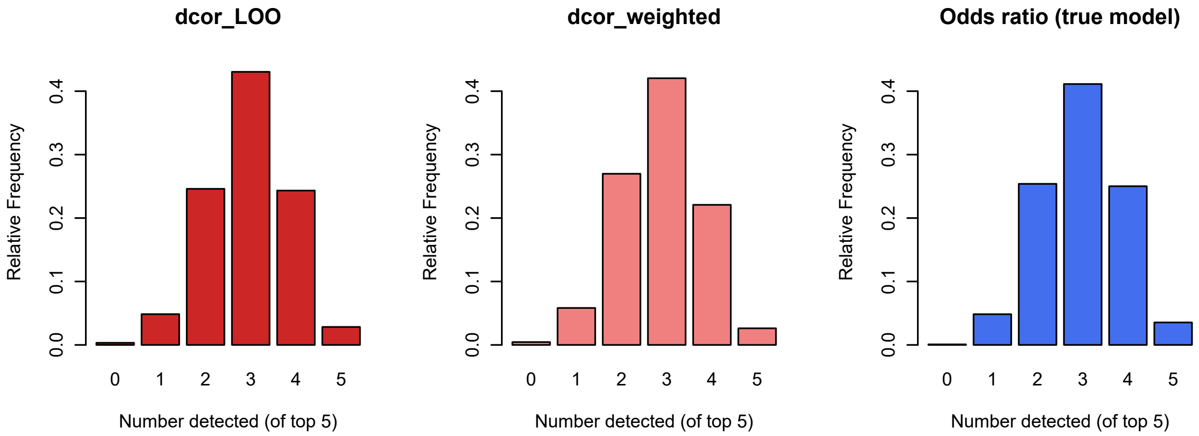

Figure S10: Histogram showing the relative frequencies of the number of top five 'true' cell types a follow-up method is able to detect from simulated data sets (Section 3.1). The $dcor_{LOO}$ method uses the largest values of $dcor_{LOO}$ (Equation 16) to find top cell types, while the $dcor_{weighted}$ method uses the largest weights (Equation 19). The odds ratio method uses the largest values of the sample odds ratio. The odds ratio method is the 'true model' in the sense that the 'true top five' are determined using the true odds ratios.

CD4 + T cells	
1	CD4 + Naïve T cells
2	CD4 + Central memory T cells
3	CD4 + TEMRA
4	CD4 + Effector memory T cells
CD8 + T cells	
1	CD8 + Naïve T cells
2	CD8 + Central memory T cells
3	CD8 + TEMRA
4	CD8 + Effector memory T cells
Non T Non B cells	
1	CD45RA-CD56-
2	CD45RA-CD56+ NK
3	CD11c + Myeloids
4	HLADR + CD11c- Monocytes
5	CD56dim CD16 + NK
6	CD56dim NK
7	CD56hi NK
8	CD14hi Monocytes
9	CD16hi Monocytes
10	CD14- CD16-
11	pDCs
12	mDCs
CD19 + B cells	
1	CD27hi B cells
2	CD27lo B cells
3	IgM + IgD + Pre-switched
4	IgM + IgD - IgM Memory
5	IgM - IgD + c-delta class switched
6	IgM - IgD - Switched Memory
7	IgM + IgD + Mature Naive
8	IgM - IgD + Immature Naive
9	IgM - IgD + Anergic B cells
10	IgM - IgD – Atypical memory

Figure S11: A complete list of cell type names for study 2 (corresponds to Figure 1 in the main manuscript)

S5 A second data analysis example

This analysis is based on data collected from an ongoing study conducted by the authors (study 2). Peripheral whole blood sample was collected from SLE patients and age and sex-matched controls. Peripheral blood samples were fixed (and red blood cells lysed) either immediately after collection (T0); or after incubation at 37C with a protein transport inhibitor cocktail (T6). Lysed/fixed cells were stored at $-80°C$, and were thawed on the day of barcoding and staining. To decrease technical variability, palladium isotopes were used in different combinations for mass tag barcoding of separate samples, pooled in sets of 20, surface stained in a single tube with a metal-labeled antibody panel. Barcoding methodology was adapted from Zunder *et al.* (2015). Protocols for intracellular cytokine staining (ICS) assays were adapted from previous studies in O'Gorman *et al.* (2017). Individual samples were barcoded to reduce technical variability, and data were also batch adjusted (Schuyler *et al.*, 2019). Clinical data including clinical laboratory parameters (complete blood cell counts, autoantibodies, chemistry panels, etc), clinical disease manifestations identified on physical exam, imaging studies, and disease severity scores (SLE disease activity index, SLEDAI) were recorded from every participant and every study timepoint (Bombardier *et al.*, 1992; Touma *et al.*, 2011). Nephritis diagnosis, to be used as a covariate, was based on renal biopsy pathology evaluation (Schwartz *et al.*, 2014).

Although questions similar to study 1 are relevant for this study too (e.g. SLE vs healthy control comparison), in this paper, for the purpose of demonstration of the application of CODAK for a continuous predictor, we focused on the association between the cell-type abundance and the SLEDAI score, a continuous predictor. Since we are interested in the disease activity (SLEDAI score), we only used the data from the SLE patients at T0. Application of CODAK on this data resulted in a p-value = 0.2794 showing that there is no significant association of the cell type compositions with the SLEDAI score. We also conducted analysis while adjusting for the effect of the binary covariate whether an SLE patient had nephritis or not. CODAK-sk and CODAK-alr both resulted in statistically non-significant outcomes (p-value = 0.3914 and 0.4298, respectively).

S6 Proof of the result in Equation 18

To show:

$$\underset{c}{\operatorname{argmin}}\ d(P_{-c}^{(i)}, P_{-c}^{(j)}) = \underset{c}{\operatorname{argmax}} \left| \ln\left[\frac{P_{ic}}{P_{jc}}\right] - \ln\left[\frac{g(P^{(i)})}{g(P^{(j)})}\right] \right|. \tag{S3}$$

Proof. Note that for this proof we can ignore the closure operator in the expression

$$P_{-c} = \mathcal{C}(P_1, P_2, ..., P_{c-1}, P_{c+1}, ..., P_q)$$

due to the scale invariance property of Aitchison Distance (AD).

It is easy to see that the AD between the two compositions $P^{(i)}$ and $P^{(j)}$ can be written as:

$$d(P^{(i)}, P^{(j)}) = \sum_{r=1}^{q} (V_r - \bar{V})^2,$$

where $V_r = \ln\left[\frac{P_{ir}}{P_{jr}}\right]$, by realizing that

$$\bar{V} = \frac{1}{q}\sum_{r=1}^{q} \ln\left[\frac{P_{ir}}{P_{jr}}\right] = \ln\left[\frac{g(P^{(i)})}{g(P^{(j)})}\right].$$

In the light of this, Equation S3 becomes

$$\underset{c}{\text{argmin}} \sum_{r\neq c}(V_r - \bar{V}_{-c})^2 = \underset{c}{\text{argmax}} \left|V_c - \bar{V}\right|, \tag{S4}$$

where $\bar{V}_{-c} = \frac{1}{q-1}\sum_{r\neq c} V_r$.

Now,

$$\begin{aligned}
\sum_{r\neq c}(V_r - \bar{V})^2 =& \sum_{r\neq c}(V_r - \bar{V}_{-c})^2 + q(\bar{V} - \bar{V}_{-c})^2 \\
\Rightarrow \sum_{r\neq c}(V_r - \bar{V}_{-c})^2 =& \sum_{r\neq c}(V_r - \bar{V})^2 - q(\bar{V} - \bar{V}_{-c})^2 \\
=& \sum_{r=1}^{q}(V_r - \bar{V})^2 - (V_c - \bar{V})^2 - q(\bar{V} - \bar{V}_{-c})^2 \\
=& \sum_{r=1}^{q}(V_r - \bar{V})^2 - (V_c - \bar{V})^2 - \frac{q}{(q-1)^2}(V_c - \bar{V})^2
\end{aligned}$$

Note that the two rightmost terms in this last equation are both increasing functions of $|V_c - \bar{V}|$. This completes the proof. □

S7 Proof of the fact that alr and clr methods lead to the same result for covariate adjustment

Let us use the notations Y_{alr} and Y_{clr} to denote the alr-transformed and clr-transformed compositional vectors. Therefore,

$$Y_{alr} = \left[\ln\left(\frac{P_1}{P_q}\right), \ln\left(\frac{P_2}{P_q}\right), ..., \ln\left(\frac{P_{q-1}}{P_q}\right)\right],$$

and

$$\begin{aligned}
Y_{clr} &= \left[\ln\left(\frac{P_1}{g(P)}\right), \ln\left(\frac{P_2}{g(P)}\right), ..., \ln\left(\frac{P_q}{g(P)}\right)\right] \\
&= (k1_n^{'} + Y_{alr},\ k),
\end{aligned}$$

where $k = \ln(\frac{P_q}{g(P)})$.

In matrix notations, for n observations,

$$\mathbf{Y_{clr}} = (k1_n1_n^{'} + \mathbf{Y_{alr}},\ k1_n).$$

Now, the residualized matrices can be written as

$$\begin{aligned}\mathbf{Y_{alr}}(Z) &= (I - H_Z)\mathbf{Y_{alr}} \\ &= R_Z\mathbf{Y_{alr}}, \quad \text{say,}\end{aligned}$$

and

$$\begin{aligned}\mathbf{Y_{clr}}(Z) &= R_Z\mathbf{Y_{clr}} \\ &= kR_Z1_n1_n^{'} + R_Z(\mathbf{Y_{alr}},\ 0_n), \quad [\ 0_n \text{ is the vector of zeros }] \\ &= kR_Z1_n1_n^{'} + (R_Z\mathbf{Y_{alr}},\ 0_n)\end{aligned}$$

Transforming back to the compositions,

$$\begin{aligned}\mathbf{P_{clr}}(Z) &= \mathcal{C}[\exp(kR_Z1_n1_n^{'}).\ \exp(R_Z\mathbf{Y_{alr}},\ 0_n)] \\ &= \mathcal{C}[\exp(R_Z\mathbf{Y_{alr}},\ 0_n)] \qquad [\text{ Since } \ \mathcal{C}(cX) = \mathcal{C}(X) \text{ for any constant } c\ ] \\ &= \mathcal{C}[\exp(R_Z\mathbf{Y_{alr}}),\ 1_n)] \\ &= \mathbf{P_{alr}}(Z).\end{aligned}$$

Hence the proof.

References

Anderson, M. J. (2001). A new method for non-parametric multivariate analysis of variance. *Austral ecology*, **26**(1), 32–46.

Anderson, M. J. (2014). Permutational multivariate analysis of variance (permanova). *Wiley statsref: statistics reference online*, pages 1–15.

Anderson, M. J. and Legendre, P. (1999). An empirical comparison of permutation methods for tests of partial regression coefficients in a linear model. *Journal of statistical computation and simulation*, **62**(3), 271–303.

Bombardier, C., Gladman, D. D., Urowitz, M. B., Caron, D., Chang, C. H., Austin, A., Bell, A., Bloch, D. A., Corey, P. N., Decker, J. L., *et al.* (1992). Derivation of the sledai. a disease activity index for lupus patients. *Arthritis & Rheumatism: Official Journal of the American College of Rheumatology*, **35**(6), 630–640.

Freedman, D. and Lane, D. (1983). A nonstochastic interpretation of reported significance levels. *Journal of Business & Economic Statistics*, **1**(4), 292–298.

Hua, W.-Y. and Ghosh, D. (2015). Equivalence of kernel machine regression and kernel distance covariance for multidimensional phenotype association studies. *Biometrics*, **71**(3), 812–820.

Kennedy, P. E. and Cade, B. S. (1996). Randomization tests for multiple regression. *Communications in Statistics-Simulation and Computation*, **25**(4), 923–936.

Manly, B. F. (2018). *Randomization, bootstrap and Monte Carlo methods in biology*. chapman and hall/CRC.

O'Gorman, W. E., Kong, D. S., Balboni, I. M., Rudra, P., Bolen, C. R., Ghosh, D., Davis, M. M., Nolan, G. P., and Hsieh, E. W.-Y. (2017). Mass cytometry identifies a distinct monocyte cytokine signature shared by clinically heterogeneous pediatric sle patients. *Journal of autoimmunity*, **81**, 74–89.

Pan, W. (2011). Relationship between genomic distance-based regression and kernel machine regression for multi-marker association testing. *Genetic epidemiology*, **35**(4), 211–216.

Schuyler, R. P., Jackson, C., Garcia-Perez, J. E., Baxter, R. M., Ogolla, S., Rochford, R., Ghosh, D., Rudra, P., and Hsieh, E. W. (2019). Minimizing batch effects in mass cytometry data. *Frontiers in immunology*, **10**, 2367.

Schwartz, N., Goilav, B., and Putterman, C. (2014). The pathogenesis, diagnosis and treatment of lupus nephritis. *Current opinion in rheumatology*, **26**(5), 502.

Touma, Z., Urowitz, M., Ibañez, D., and Gladman, D. (2011). Sledai-2k 10 days versus sledai-2k 30 days in a longitudinal evaluation. *Lupus*, **20**(1), 67–70.

Winkler, A. M., Ridgway, G. R., Webster, M. A., Smith, S. M., and Nichols, T. E. (2014). Permutation inference for the general linear model. *Neuroimage*, **92**, 381–397.

Zhao, N., Chen, J., Carroll, I. M., Ringel-Kulka, T., Epstein, M. P., Zhou, H., Zhou, J. J., Ringel, Y., Li, H., and Wu, M. C. (2015). Testing in microbiome-profiling studies with mirkat, the microbiome regression-based kernel association test. *The American Journal of Human Genetics*, **96**(5), 797–807.

Zunder, E. R., Finck, R., Behbehani, G. K., El-ad, D. A., Krishnaswamy, S., Gonzalez, V. D., Lorang, C. G., Bjornson, Z., Spitzer, M. H., Bodenmiller, B., *et al.* (2015). Palladium-based mass tag cell barcoding with a doublet-filtering scheme and single-cell deconvolution algorithm. *Nature protocols*, **10**(2), 316.
